# Freeze–thaw process of soil between two piles as monitored by piezoelectric ceramic sensor

**DOI:** 10.1038/s41598-023-32929-2

**Published:** 2023-04-07

**Authors:** Daopei Zhu, Zhongyong Lai, Zhangli Wang

**Affiliations:** 1 School of Civil and Surveying & Mapping Engineering, Jiangxi University of Science and Technology, Ganzhou, 341000 China; 2 Gansu Academy of Building Research (Group) Corporation Limited, Lanzhou, 730070 China

**Keywords:** Engineering, Civil engineering

## Abstract

As the mechanical properties of soil are affected by the moisture content, diameter of soil particles, and the soil temperature, we used piezoelectric ceramic sensors to monitor the freeze–thaw cycle of different soils at different temperatures and different moisture content. By studying the energy attenuation of stress waves propagating in freezing–thawing soil, its mechanical strength was determined. The results showed that the duration of freeze–thaw process was related to soil type and initial water content. For the same water content and larger soil particle size, the received signal amplitude and energy are larger. For the same soil type and higher water content, the received signal amplitude and energy are stronger. This study provides a feasible monitoring method for infrastructure construction in areas with complex geological conditions, such as Qinghai-Tibet frozen soil.

## Introduction

The interaction between frozen soil environment and engineering construction is of great significance when long-term construction is carried out in low-temperature regions, and the influencing factors of frozen soil strength^1^are also of great importance. From a microscopic point of view, the strength of frozen soil layer is composed of three types of linkages: molecular linkages (van der Waals forces), structural bond and ice-cement linkages^2^, among which ice-cement linkages plays a dominant role. Regarding the reinforcement mechanism of frozen soil. Ting et al.^3^ inferred that in frozen soil, the soil acts as a reinforcement in ice, thus improving the overall strength of the frozen soil. Environmental temperature^4^, soil type^5^ and water content^6^ were the first factors to be considered in the study of permafrost strength. Chamberlain et al.^7^ conducted freezing experiments on saturated sand and silty sand, and found that different soil types led to changes in frozen soil strength.

In the process of monitoring the change of frozen soil strength, it is necessary to consider the basic conditions of frozen soil environment and choose corresponding monitoring methods and means. When monitoring the water content of frozen soil: Zhang et al.^8^ used thermal pulse probe method to measure the moisture content of frozen soils. Schwank et al.^9^ used microwave technology to monitor soil moisture. Zhao et al.^10^ used AMSR-E passive microwave imaging to monitor the soil moisture content based on microwave technology. In order to improve the accuracy of monitoring, Gao et al.^11^ used AMSR-E and AMSR2 algorithms to evaluate soil freezing and thawing conditions, which improved the effect of monitoring frozen soil strength. When measuring large-area frozen soil conditions, Zhang et al.^12^ used a satellite to monitor and analyze soil water content changes, which could be used to monitor water content in ground soil over a large area. Mavrovic et al.^13^ used two different instruments to measure the dielectric constant and proposed that the improvement of the dielectric model could greatly improve the benefit of satellite freeze–thaw detection.

Piezoelectric ceramics are being promoted for use in various fields. Piezoelectric materials^14^ can directly convert mechanical energy to electric energy. Hence, their operation and principle are very simple and well understood. On this basis, improved piezoelectric ceramic materials^15^ can be used in more applications. Tseng et al.^16^ described the application and development of lead zirconate titanate (PZT) ceramics. Schulz et al.^17^ used piezoceramic patches for monitoring and active control to understand the health of composite structures. Song et al.^18^ used piezoelectric ceramics and wireless sensor networks to monitor the health of wind-turbine blades. Liu et al.^19^ conducted an exploratory study on seepage monitoring of concrete structures using a piezoelectric ceramic intelligent aggregate.

The use of piezoelectric ceramics to change the strength of frozen soil under different conditions such as temperature, moisture content and porosity. With the widespread application of piezoelectric ceramics, researchers have gradually recognized the advantages of using piezoelectric ceramics to study the freeze–thaw cycle of frozen soil^20^. Kong et al.^21^ used piezoelectric ceramic intelligent aggregate to monitor the soil freeze–thaw process, and achieved good results. Zhang et al.^22^ used the electromechanical impedance method of PZT to monitor the freeze–thaw process in soil. Piezoelectric ceramics actively monitor the building structure without damaging the building structure, which is more suitable for long-term monitoring of the building structure in the project.

In this study, two piezoceramic sensors were used to transmit and receive signals. The waveform and amplitude of the signal transmitted by the signal transmitter were the same, and the energy consumed by the stress wave propagating in different soils was different. In the freeze–thaw cycle of soil, the strength of frozen soil was determined by studying the influence of soil type and moisture content on the energy attenuation of the stress wave. The results show that this method is effective to study the freeze–thaw properties of soil.

## Monitoring methods

### Soil monitoring method based on piezoelectric ceramic sensor

The freeze–thaw cycles of clay and medium sand were monitored using a piezoelectric ceramic sensor. In this experiment, two piezoelectric ceramic sensors were used, among which one was connected to a signal transmitter to transmit stress wave signals and the other was connected to a signal receiver to receive stress wave signals. The propagation of stress waves is affected by the medium between the transmitter and receiver. In the freeze–thaw cycle of soil, water or ice constantly undergoes a phase transformation. Water or ice combines with soil particles to form various soil microstructures, which affect the mechanical properties of frozen soil. The stress wave signals have different responses due to the change in soil medium in the freeze–thaw cycle, and the phase transformation process of water and ice is as follows:

First item; When there is only liquid water in the soil, water is a damping factor, and the propagation of stress waves is significantly affected.

Second item; The water molecules in soil are in a state of solid–liquid coexistence^23^. At this stage, with an increase in ice content, the stress wave propagates better in the soil, and if the soil has a high moisture content, its transformation process is relatively longer.

Thirdly item; All the water in the soil exists in a solid state: in this state, the stiffness of the soil will increase as a whole, and the soil in this state is the best state for stress wave propagation.

In this study, we investigated the influence of soil type and initial water content on the stress wave response of soil during the freeze–thaw cycle, and attempted to estimate the change in the mechanical properties of frozen soil during the freeze–thaw process using a piezoelectric ceramic sensor.

### Basic principles of time domain analysis

The stress-wave energy transmission through the soil specimen is sensitively correlated to the soil mechanical properties, and hence the energy response recorded at the sensor can be used as an indicator to describe the soil mechanical properties and even the freeze–thaw situation^24^. The signal recorded on the sensor is decomposed into a group of frequency bands based on a time-domain analysis. The total energy of the stress wave of the soil sample can be calculated by accumulating all the energy of the signals in different time domains. In time-domain analysis, the statistical eigenvalues include the maximum value, minimum value, average value, mean square value, signal amplitude variance, and signal energy and power. The energy statistics of the signals are the most widely used parameters. The equation to calculate the signal energy is.

Where *X*_*i*_ represents a set of discrete data signals sampled by the sensor in a specific sampling time. In *X*_*ij*_, *j* represents the sampling time point of the sensor value at the same time point. The total sampling points are *m* in each sampling duration.

1 $${\text{x}}_{i} = (x_{i1} , \, x_{i2} , ..., x_{im} ),$$

2 $$E_{i} = \left\| {{\text{x}}_{i} } \right\|^{2} = \sum\limits_{j = 1}^{m} {x_{ij} } (i = 1,2...).$$

The attenuation of signal energy is denoted by *H*_*i*_, which is defined as

3 $${H}_{i}=\frac{{E}_{i}}{{E}_{0}},{E}_{0}=m{x}_{0}^{2},$$

where, $x_{0}$ and $E_{0}$ are the amplitude and energy, respectively, of the signal sent by the signal generator. *H*_*i*_ can be used as an index to describe the freezing–thawing process of soil, and is a potential index of the mechanical properties of the soil, such as the soil strength.

## Experimental setup

### Test, sample and data acquisition system

Two soil types, i.e. clay soil and medium sand were used in these experiments. The particles of clay soil were very fine (diameters ranging from 0.005 mm to 0.05 mm) and had high plasticity and low permeability. In addition, the particle size of medium sand was between 0.35 mm and 0.5 mm, with low plasticity and high permeability.

**Figure 1:**
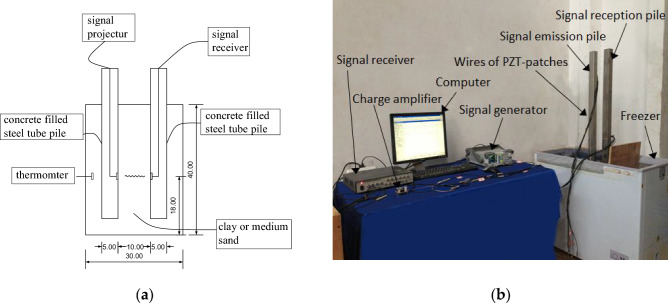
Schematic diagram of experimental apparatus. (**a**) Sample device. (**b**) Layout of experimental equipment.

Two piezoelectric ceramic sensors, 10 cm apart, and a thermometer were inserted into each sample. The moisture content of the clay sample was approximately 30%, and the moisture contents of medium-sand samples were 10%, 15%, and 20%. As shown in Fig. 1b, Two square cement tubes with a cross section side length of 5 cm were inserted into the test soil sample. Among them, on both sides of every 9 cm with PZT piezoelectric patches, launcher energy wave from a signal through the signal launch pile after pile soil specimen to reception, by the charge amplifier amplification energy indicator. Finally, the data was collected by the signal receiver. As shown in Fig. 1a, the length, width, and height of the test chamber were 30 cm, 20 cm and 40 cm, respectively. The square wave emitted by the signal transmitter had a frequency of 1000 Hz and an amplitude of 100,000 mV.

### Experimental procedure

To simulate the freezing environment, a controllable-temperature refrigerator was used to cool the sample. At a room temperature of 26 °C, clay soil and medium sand were selected to use electric heating dryer to dry their original moisture; clay soil was used to produce test soil samples with moisture contents of 10%, 15%, 20%, 25%, and 30%, and medium sand was used to produce test soil samples with moisture contents of 10%, 15%, and 20%. A total of 8 groups of soil samples were made, Specific parameters of soil sample production are shown in Table 1. First, two concrete piles with piezoelectric ceramic sensors are vertically placed in the test box. At the same time, the test box is filled with soil samples and compacted, and then put into the refrigerator, the temperature of the soil samples is reduced to − 20 °C; Then the temperature of the refrigerator is kept at − 20 °C, the signal generator continuously transmits signals to the soil, and the receiver continuously receives signals. A total of more than eight groups of operation experiments were conducted and experimental data were recorded.

**Table 1:** Test the material properties of the soil sample.

Soil sample name	Soil sample type	Moisture	Density (g/cm^3^)
10% moisture clay soil	Clay soil	10%	1.73
15% moisture clay soil	Clay soil	15%	1.85
20% moisture clay soil	Clay soil	20%	1.93
25% moisture clay soil	Clay soil	25%	2.04
30% moisture clay soil	Clay soil	30%	2.08
10% moisture medium sand	Medium sand	10%	1.65
15% moisture medium sand	Medium sand	15%	1.73
20% moisture medium sand	Medium sand	20%	1.81

## Experimental results and analysis

### Freezing process of specimens in freeze–thaw cycle

**Figure 2:**
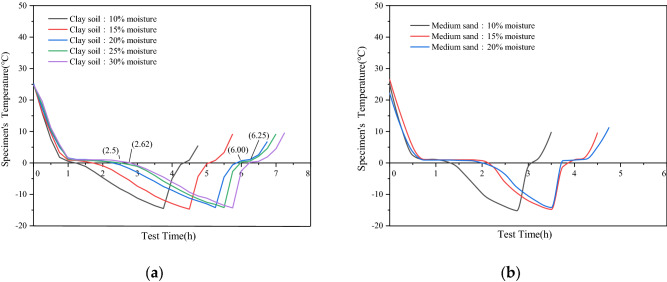
Temperature change of samples with different moisture content. (**a**) Temperature change of clay samples with different moisture content;(**b**) Temperature change of medium-sand samples with different moisture content.

**Figure 3:**
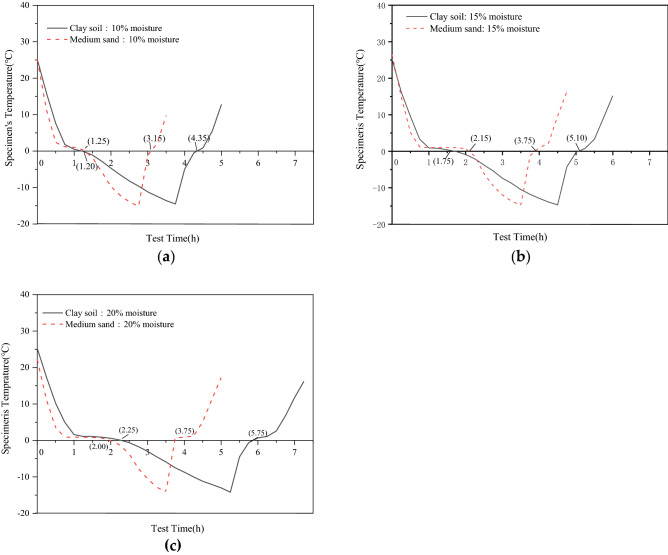
Temperature change of samples with the samebut different moisture content. (**a**) Two samples, each with a moisture content of 10%; (**b**) Two samples with a moisture content of 15%; (**c**) Two samples with a water content of 20% each.

The moisture content of the clay samples was 10%, 15%, 20%, 25%, and 30%. When the temperature dropped to 0 °C, the testing times were approximately 72 min, 105 min, 120 min, 150 min, and 156 min, respectively, as shown in Figs. 2 and 3. The moisture content of the medium sand samples was 10%, 15% and 20% respectively, and the test times were approximately 75 min, 129 min, and 135 min, respectively, when the temperature dropped to 0 °C, as shown in Fig. 3. Then, the temperature was lowered from 0 °C to − 15 °C, and the test times of the five clay samples were 186 min, 183 min, 210 min, 210 min, and 218 min, respectively, as shown in Figs. 2a and 3. The test times for the three medium sand samples were 117 min, 99 min, and 105 min, respectively, as shown in Figs. 2b and 3. As shown in Fig. 3, for higher initial moisture content of the sample, greater amount of ice was contained in the corresponding sample after freezing. Thus, the freezing time was longer.

A comparison of the two different samples with moisture content of 10%, 15%, and 20% shows that the freezing speed of the medium sandy soil samples is faster than that of the clay soil samples, as shown in Fig. 3. Medium sandy soil samples have large pores and strong permeability. Therefore, the water flow in the medium sandy soil is not subject to the capillary action between water and soil. In contrast, the capillary diameter of clay significantly affects the water flow. For greater fluidity of water, the freezing speed of the soil sample is faster^25,26^.

### Monitoring of specimens during freeze–thaw cycles

**Figure 4:**
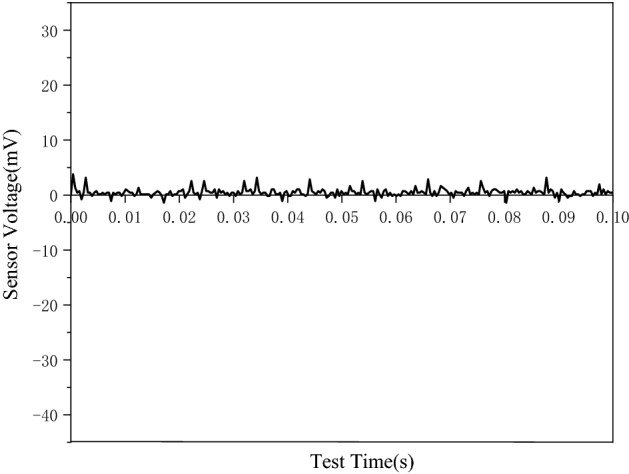
Signal response diagram under the influence of environmental noise.

**Figure 5:**
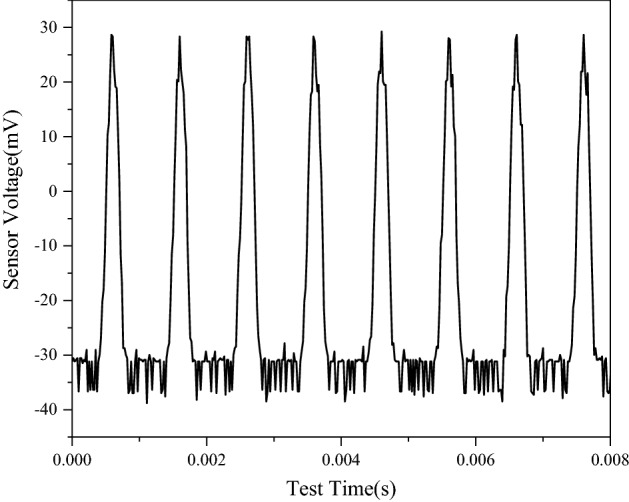
Sensing voltage variation during frozen soil measurement.

In order to eliminate the interference of fluctuations caused by external magnetic field in the experimental study, external energy waveform is collected when the launching pile is not working, as shown in Fig. 4. At this point, the soil sample between the two piles was clay with a moisture content of 15% and at a temperature of 0 °C. The signal received by the signal receiving pile when the signal generator was turned on, is shown in Fig. 5.Thus it is evident that the signal received by the signal-receiving pile is indeed the signal emitted by the signal-transmitting pile.

**Figure 6:**
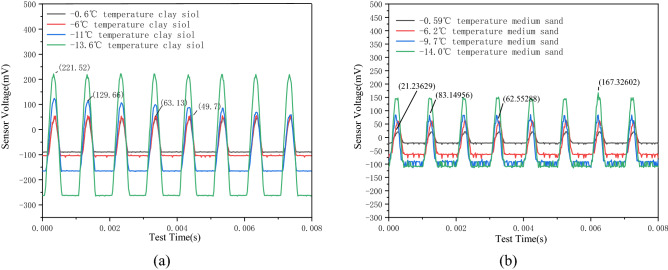
Sensing voltage changes of samples at different temperatures. (**a**) Sensor voltage variation diagram of clay samples at different temperatures. (**b**) Sensor voltage variation diagram of medium sand samples at different temperatures.

As shown in Fig. 6a, the signal amplitudes of the clay samples with an initial water content of 20% at four different temperatures (− 0.6 °C, − 6 °C, − 11 °C, and − 13.8 °C) are 49.7 mV, 63.13 mV, 129.66 mV, and 221.52 mV, respectively. Meanwhile, as shown in Fig. 6b, the medium-sand sample with an initial water content of 20% reaches an amplitude of 21.23 mV, 62.55 mV, 83.14 mV, and 167.32 mV during freezing at four different temperatures (− 0.6 °C, − 5.9 °C, − 9.7 °C, and − 14.0 °C). Hence, with a decrease in temperature, the amplitude of the signal received by the two samples increases. Simultaneously, the strength of the soil also increases, and thus, more signal wave energy can be received by the receiver. Therefore, the strength of the soil is reflected by the amplitude of the signal.

**Figure 7:**
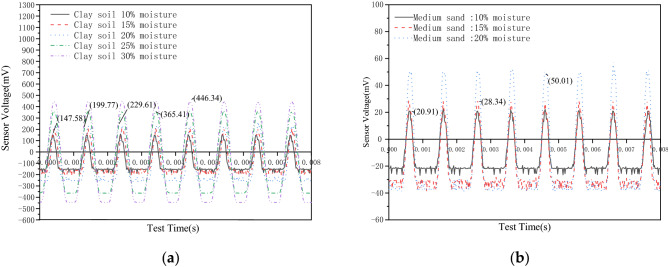
Sensing voltage changes of the two samples with different moisture content; (**a**) Sensing voltage variation diagram of clay samples with different moisture content; (**b**) Changes in the induced voltage diagram of sand samples with different moisture content.

At − 13.6 °C, the signal amplitudes of the five clay samples are 147.58 mV, 199.77 mV, 229.61 mV, 365.41 mV, and 446.34 mV, respectively, as shown in Fig. 7a. Meanwhile, the signal amplitudes of the three medium-sand samples at − 14 °C are 20.91 mV, 28.34 mV, and 50.01 mV, respectively, as shown in Fig. 7b. Based on this analysis, the amplitude of the signal received by the sensor increases with an increase in the initial water content of the sample during freezing. During freezing, clay or medium-sand particles combine with ice particles to form a hard soil sample. With an increase in the initial water content, more ice particles are formed during the freezing process, which can combine with more soil particles to form a harder soil sample. At this time, the mechanical strength of the soil sample is continuously enhanced. Therefore, the amplitude of the sample signal received by the sensor when the soil sample is frozen is positively correlated with its water content.

**Figure 8:**
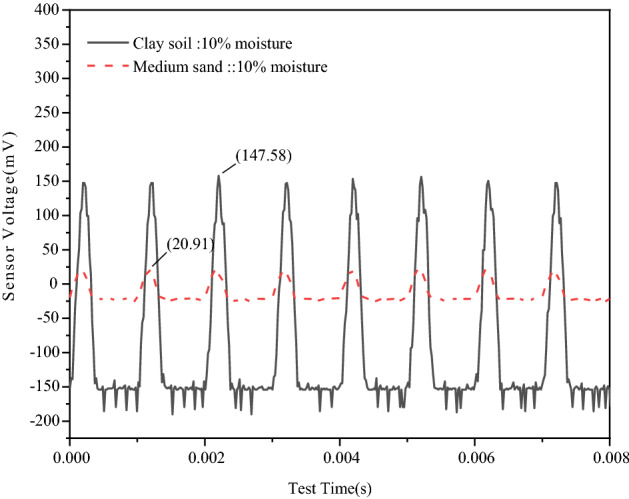
Energy waveform of different samples with the same moisture content.

At − 13.8 °C, when the moisture content of both the clay sample and medium-sand sample is 10%, the signal amplitudes received by the sensors in the two samples are 147.58 mV and 20.91 mV, respectively, as shown in Fig. 8. It is evident from the data that the amplitude of the signal received by the clay sample is significantly larger than that by the medium-sand sample. In the freeze–thaw cycle, the particles in the clay sample are more compact than those in the medium sand sample, and the stress wave is more stable in the transmission process. The signal amplitude of the clay sample is higher than that of the medium sand sample. Hence, the signal amplitude value of the sample is also positively correlated with the strength of the sample^27^.

### Influence of soil type and initial water content

**Figure 9:**
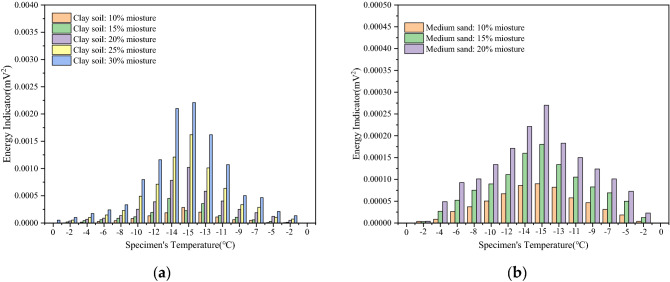
Energy indicator diagram of samples with different moisture content. (**a**) Energy indicator of clay samples with different moisture content; (**b**) energy indicator of sand samples with different water content.

**Figure 10:**
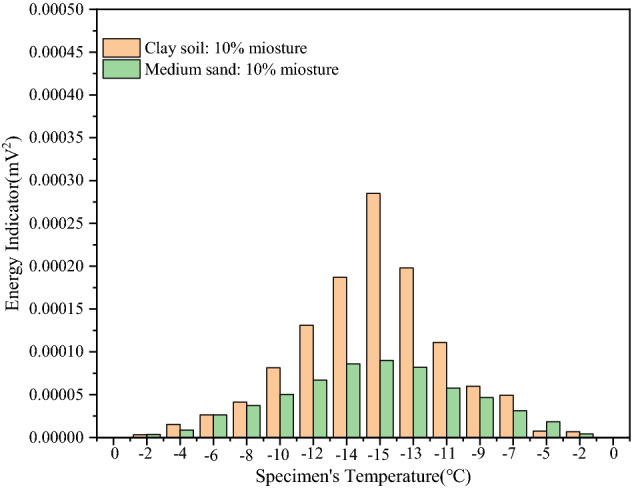
Energy indicator diagram of different samples with the same water content.

To monitor the degree of attenuation of the stress wave in the propagation process, the corresponding energy indicator was calculated for the freeze–thaw process of different soils; Where, the energy indicator is expressed by the square value of signal amplitude in the statistical characteristic value. Using this index, the soil type and initial water content were studied, and the influence of water content on the freezing and thawing processes of the soil was analyzed. As shown in Fig. 9, during the freezing process, the energy indicator of the soil continues to increase while the temperature continues to decrease. This is consistent with the increase in the strength of the frozen soil when the temperature decreases. In the entire freeze–thaw cycle test, the temperature decreases to the lowest value, the strength of the clay and medium-sand samples also reach the highest values at the same time, and the energy indicator reaches its maximum. Subsequently, the soil temperature gradually increases, the ice particles in the sample slowly melt, and the energy indicator of the signal wave decreases, as shown in Fig. 9. This shows that the strength of the soil samples decreases with an increase in temperature. The energy indicator also increases with an increase in the initial water content. According to the analysis, when the initial moisture content of the sample is higher, more ice particles are generated in the freezing process of the soil, and the ice particles are more dense with soil particles, thus increasing the strength of the soil sample. As shown in Fig. 10, for the same initial water content, the energy received by the clay samples in the freeze–thaw cycle is higher than that received by the medium-sand samples. This indicates that the strengths of the clay samples are higher than those of the medium-sand samples in the freeze–thaw cycle. This is because, under the same water content, the soil cohesion of the clay samples is higher than that of the medium-sand samples. In consequence, the clay samples have a stronger cohesive force, resulting in a stronger energy indicator and greater strength. The experimental results show that the energy indicator used in this experiment can accurately reflect the changes in the physical characteristics of soil during freeze–thaw cycles.

## Conclusions

During the freeze–thaw process, the properties of soil can be determined by monitoring the signal strength. With a decrease in temperature, the water in the soil gradually freezes, and the strength of the soil increases, thus reducing the energy attenuation in the stress wave propagation process. This study focuses on the determination of soil strength based on the strength of energy transfer. In the freeze–thaw cycle, different water contents and particle sizes lead to different microstructures and mechanical properties. When the initial water content of the sample is the same, for larger particle size, the signal energy received by the signal-receiving pile is stronger. When the same soil type, with higher initial water content of the sample, the signal energy received by the signal-receiving pile is stronger. In summary, piezoelectric ceramics can effectively monitor the characteristics of frozen soil based on the energy index of signal wave energy attenuation during propagation. In combination with the active monitoring effect of piezoelectric ceramics, it can be more widely used in the monitoring of frozen soil characteristics.

## Data Availability

The experimental data used to support the findings of this study are included within the article.

## References

[1] Martin C, Kim Y-C, Park J-B (2009). The influence of temperature and cycles on acoustic and mechanical properties of frozen soils. KSCE J. Civ. Eng..

[2] Milana JP (2016). Molards and their relation to landslides involving permafrost failure. Permafr. Periglac. Process..

[3] Ting JM, Torrence Martin R, Ladd CC (1983). Mechanisms of strength for frozen sand. J. Geotech. Eng..

[4] Yang Z, Still JB, Ge X (2015). Mechanical properties of seasonally frozen and permafrost soils at high strain rate. Cold Reg. Sci. Technol..

[5] Harlan RL (1973). Analysis of coupled heat-fluid transport in partially frozen soil. Water Resour. Res..

[6] Suzuki S (2004). Dependence of unfrozen water content in unsaturated frozen clay soil on initial soil moisture content. Soil Sci. Plant Nutr..

[7] Chamberlain E, Groves C, Perham R (1972). The mechanical behaviour of frozen earth materials under high pressure triaxial test conditions. Geotechnique.

[8] Yinsuo Z, Treberg M, Carey SK (2011). Evaluation of the heat pulse probe method for determining frozen soil moisture content. Water Resour. Res..

[9] Schwank M, Stahli M, Wydler H, Leuenberger J, Matzler C, Fluhler H (2004). Microwave L-band emission of freezing soil. IEEE Trans. Geosci. Remote Sens..

[10] Zhao T, Zhang L, Jiang L, Zhao S, Chai L, Jin R (2011). A new soil freeze/thaw discriminant algorithm using AMSR-E passive microwave imagery. Hydrol. Process..

[11] Gao H, Zhang W, Chen H (2018). An improved algorithm for discriminating soil freezing and thawing using AMSR-E and AMSR2 soil moisture products. Remote Sens..

[12] Tingjun Z, Barry RG, Armstrong RL (2004). Application of satellite remote sensing techniques to frozen ground studies. Polar Geogr..

[13] Mavrovic A, Lara RP, Berg A, Demontoux F, Royer A, Roy A (2021). Soil dielectric characterization during freeze–thaw transitions using L-band coaxial and soil moisture probes. Hydrol. Earth Syst. Sci..

[14] Anton SR, Sodano HA (2007). A review of power harvesting using piezoelectric materials (2003–2006). Smart mater. Struct..

[15] Piazza D, Capiani C, Galassi C (2005). Piezoceramic material with anisotropic graded porosity. J. Eur. Ceram. Soc..

[16] Tseng KK, Naidu AS (2002). Non-parametric damage detection and characterization using smart piezoceramic material. Smart Mater. Struct..

[17] Schulz MJ, Pai PF, Inman DJ (1999). Health monitoring and active control of composite structures using piezoceramic patches. Compos. B.

[18] Song G, Hua L, Bosko G, Wensong Z, Peng C, Haichang G (2013). Wind turbine blade health monitoring with piezoceramic-based wireless sensor network. Int. J. Smart Nano Mater..

[19] Liu T, Yongchao H, Dujian Z, Jun T, Bo L (2013). Exploratory study on water seepage monitoring of concrete structures using piezoceramic based smart aggregates. Smart Mater. Struct..

[20] Kong Q, Wang R, Yang ZJ, Yuqian W (2014). Seasonal ground freezing and thawing monitoring using piezoceramic based smart aggregates. Earth Space.

[21] Qingzhao K, Wang R, Song G, Yang ZJ (2014). Monitoring the soil freeze-thaw process using piezoceramic-based smart aggregate. J. Cold Reg. Eng..

[22] Zhang J, Chuan Z, Jiahao X, Jinwei J (2019). A pzt-based electromechanical impedance method for monitoring the soil freeze–thaw process. Sensors.

[23] Pardo Lara R, Berg AA, Warland J, Parkin G (2021). Implications of measurement metrics on soil freezing curves: A simulation of freeze–thaw hysteresis. Hydrol. Process..

[24] Soh CH, Tseng KK, Bhalla S, Gupta A (2000). Performance of smart piezoceramic patches in health monitoring of a RC bridge. Smart Mater. Struct..

[25] Wang B, Cao Y, Rong C, Cheng H (2022). Study on the mechanism and prevention method of frozen wall maldevelopment induced by high-flow-rate groundwater. Water.

[26] Kim SY, Hong WT, Lee JS (2018). Silt fraction effects of frozen soils on frozen water content, strength, and stiffness. Constr. Build. Mater..

[27] Kong QZ, Wang RL, Song GB (2004). Monitoring the soil freeze-thaw process using piezoceramic-based smart aggregate. J. Cold Reg. Eng..

